# 5-Methoxytryptophan Alleviates Dextran Sulfate Sodium-Induced Colitis by Inhibiting the Intestinal Epithelial Damage and Inflammatory Response

**DOI:** 10.1155/2024/1484806

**Published:** 2024-07-17

**Authors:** Yanling Wang, Jun Li, Qinyuan Yang, Zhenhang Zhu, Fang Cheng, Xiangyan Ai, Yang Liu, Dongbao Zhao, Futao Zhao, Peng Cheng

**Affiliations:** ^1^ Department of Rheumatology and Immunology Shanghai Ninth People's Hospital Shanghai Jiao Tong University School of Medicine, Shanghai, China; ^2^ Department of Rheumatology and Immunology Shanghai Tenth People's Hospital Tongji University School of Medicine, Shanghai, China; ^3^ Department of Geriatrics Shanghai Health and Medical Center, Wuxi, Jiangsu 214000, China; ^4^ Department of Rheumatology and Immunology Changhai Hospital Naval Medical University, Shanghai 200433, China; ^5^ Department of Gastroenterology Hainan West Central Hospital, 2 Fubo East Road, Danzhou, Hainan, China; ^6^ Department of Gastroenterology Shanghai Ninth People's Hospital Shanghai Jiao Tong University School of Medicine, No. 639 Zhizaoju Road, Shanghai, China

## Abstract

**Background:**

Colitis is a refractory intestinal inflammatory disease significantly affecting the quality of a patient's life and increasing the risk of exacerbation. The primary factors leading to colitis encompass infections, insufficient blood flow, and the buildup of collagen as well as white blood cells. Among various available therapeutics, 5-methoxytryptophan (5-MTP) has emerged as one of the protectants by inhibiting inflammatory damage. Nonetheless, there is no report on the role of 5-MTP in the treatment of colitis.

**Materials and Methods:**

To verify the anti-inflammatory effect of 5-MTP *in vivo*, we first constructed mouse model with dextran sulfate sodium-induced colitis. Furthermore, the macrophage infiltration and release of inflammatory factors through western blot (WB) and hematoxylin–eosin staining analyses were examined. Intestinal epithelial cell tight junction damage and apoptosis were investigated by WB analysis, immunofluorescence, and terminal deoxynucleotidyl transferase dUTP nick end labeling staining. Finally, we examined the generation of cellular inflammation and analyzed the influence of 5-MTP on M1 polarization at the cellular level.

**Results:**

This study initially confirmed that 5-MTP possessed an excellent therapeutic effect on colitis. 5-MTP inhibits macrophage infiltration and the generation of inflammatory factors. In addition to its effects on immune cells, 5-MTP significantly inhibits intestinal epithelial cell tight junction damage and apoptosis *in vivo*. Moreover, it inhibits inflammation and M1 polarization response *in vitro*.

**Conclusion:**

5-MTP counteracts excessive inflammation, thereby preventing intestinal epithelial tight junction damage. In addition, inhibition of apoptosis suggests that 5-MTP may be a potential therapeutic agent for colitis.

## 1. Introduction

Colitis is a refractory intestinal inflammatory disease, due to various causes, such as infections, loss of blood supply, and accumulation of collagen [1]. The predominant clinical symptoms include abdominal pain, which is seriously affecting the quality of life of patients [2] and increasing the risk of exacerbation [3]. While it is true that there are a range of environmental factors that commonly trigger colitis, it is important to understand that individuals with a genetic predisposition are particularly vulnerable when it comes to these environmental factors. In addition, the development of colitis is closely associated with impaired mucosal barriers and disturbed immune responses within the gastrointestinal tract [4, 5]. Various nonsteroidal anti-inflammatory drugs and steroidal hormones have been applied to treat colitis in clinical. Nonetheless, the application of these drugs leads to recurrence of colitis and results in undesired adverse effects due to the unfamiliar pathogenesis and relieving only some clinical symptoms of colitis [6, 7]. Therefore, there is an urgent need to incessantly explore safer and more effective drugs to treat colitis.The 5-methoxytryptophan (5-MTP), a product of tryptophan metabolism, has emerged as one of the potential drugs derived from the tryptophan hydroxylase pathway [8]. 5-MTP was originally named cytoprotection due to its action of protecting tissues from inflammatory damage [9, 10]. After systematic metabolomics and genetic-based analyses, 5-MTP was identified as a metabolite of tryptophan [8]. Notably, 5-MTP is often secreted from fibroblasts, endothelial cells (ECs), and epithelial cells [11]. Previous studies have indicated that 5-MTP could substantially control inflammation by blocking p38 and nuclear factor NF-*κ*B activation, thereby attenuating immune cell infiltration [12, 13]. In addition, 5-MTP could protect endothelial barrier function, control molecular adhesion expression, inhibit macrophage migration and activation, and release proinflammatory cytokines and chemokines [13]. Innate anti-inflammatory molecule could offer anticancer effects to a certain extent. However, there is no report concerning the role of 5-MTP in the treatment of this common intestinal inflammation. Particularly, it is required to explore the potential of 5-MTP as a safer and more effective than traditional treatments.

Based on these considerations, we decided to construct the mouse model of 2,4,6-trinitrobenzenesufonic acid (TNBS)-, DSS-induced colitis to investigate the critical role of 5-MTP in controlling the inflammatory response. For the first time, the results of the study showed that 5-MTP possessed an excellent therapeutic effect in a DSS-induced colitis model, broadening the application of its anti-inflammatory effect. Our findings further demonstrated that 5-MTP inhibited the macrophage infiltration and generation of inflammatory factors. In addition to its effects on immune cells, the 5-MTP combated excessive inflammation and prevented intestinal epithelial tight junction damage and apoptosis. There was a notable decrease in the levels of IL-1*β*, IL-6, TNF-*α*, and iNOS, which indicated that 5-MTP effectively reduced the expression of relevant pro-inflammatory cytokines and M1 polarization response in RAW264.7 cells and showed its potential as a therapeutic drug for colitis.

## 2. Materials and Methods

### 2.1. Animal Model

Total 20 male C57BL/6 mice (Shanghai Center for Model Organisms, Shanghai, China) of specific pathogen-free grade (8–10 weeks old) were selected and freely ingested water and food. Room temperature was maintained at 20−24°C and relative humidity at 40%–60%. All animals were randomly divided into four groups, mice were intragastrically administered with a 3% DSS (Solarbio Life Sciences, Beijing, China) solution for 7 days to establish an acute colitis model. After successful modeling, the treatment groups were intragastrically administered with low- (50 mg/kg/day, Sigma, MO, USA) and high-dose 5-MTP (100 mg/kg/day) solutions for 7 days, while the control group and DSS group were intragastrically administered with an equal amount of distilled water [14]. Additionally, the Disease Activity Index (DAI) was enumerated by recording weight loss, fecal diarrhea, and fecal blood. After 14 days, mice were humanely sacrificed by cervical dislocation. Colon tissue was excised for subsequent analysis.

The TNBS induction of TNBS colitis (Sigma–Aldrich, Inc., St. Louis, MO, USA) with the addition of BALB/c mice (6–8 weeks of age, 18–20 g) to 50% ethanol. Different concentrations of 5-MTP were intragastric 2 hr after intrarectal injection of TNBS. The mice were monitored daily for weight loss and disease severity using the disease activity index (DAI). At the end of the experiment, after the mice were euthanized, the colons were taken for length-measuring pathological sections.

All experimental procedures involving animals were studied and approved by the ethics committee of Shanghai Jiao Tong University School of Medicine, Shanghai, China, following the Institutional Animal Care and Use Committee (IACUC) guidelines.

### 2.2. Cell Culture and Polarization Stimulation

The RAW264.7 macrophage cell line was cultured in DMEM (HyClone, Logan, UT, USA) supplemented with 10% fetal bovine serum (Gibco, New York, USA), 100 U/ml penicillin and streptomycin (Gibco, New York, USA). The cells were maintained at 37°C in a 5% CO_2_. RAW264.7 macrophages were stimulated with LPS plus IFN-*γ* 24 hr to display the M1 macrophages phenotype.

### 2.3. Western Blot

Briefly, the tissue and cell were initially pulverized, and the homogenate was lysed with precooled radioimmunoprecipitation assay lysis buffer (Roche, Basel, Switzerland) to extract the tissue protein. Further, the total protein was detected by the Pierce BCA Protein Assay Kit (ThermoFisher Scientific, Waltham, USA). Then, the protein samples were separated by SDS-PAGE (Sigma Co., Ltd., St. Louis, USA) and transferred to polyvinylidene difluoride (PVDF, Bio-Rad, Hercules, USA) membranes. The membrane was blocked by 5% skimmed milk for 1 hr. Furthermore, the membranes were incubated with anti-CD206 (1 : 1,000), anti-CD86 (1 : 1,000), anti-Bax (1 : 2,000), anti-Bcl-2 (1 : 1,000), anti-caspase3 (1 : 1,000), anti-PARP1 (1 : 1,000), anti-IL-1*β* (1 : 000), anti-IL-6 (1 : 1,000), anti-TNF-*α* (1 : 1,000), anti-GAPDH (1 : 5,000), and anti-*β*-actin (1 : 1,000) primary antibodies (Abcam, Cambridge, UK) overnight at 4°C. After 24 hr, the membranes were washed three times and incubated with horseradish peroxidase-labeled secondary antibodies (Abcam, Cambridge, UK) for 1 hr at room temperature. Finally, the generated protein bands were visualized by exposure to enhanced chemiluminescence (Bio-Rad) luminescent solution using an imaging system (BIO-RAD Gel Doc XR). The membranes were cut horizontally.

### 2.4. Immunofluorescent Staining

Tissue sections were initially deparaffinized, rehydrated, and treated with 3% hydrogen peroxide, followed by antigen retrieval in boiling citrate buffer (0.1 M, pH 6.0). Sections were then blocked with 20% goat serum for 30 min. TUNEL staining was conducted using the ApopTag kit (Chemicon International, Temecula, USA) as per the manufacturer's instructions. For tight junction analysis, sections were immunostained for ZO-1, occludin, and claudin-2 specific mouse antibodies: ZO-1 (1 : 200), occludin (1 : 200), and claudin-2 (1 : 100) (Santa Cruz Biotechnology, Dallas, USA), incubated overnight at 4°C, followed by incubation with Alexa 594 or Alexa 488-labeled secondary antibodies (Invitrogen, Waltham, USA). Finally, the tissue sections were counterstained with DAPI (Beyotime, Shanghai, China), and the images were captured by a fluorescence microscope (CLSM, Zeiss, Oberkochen, Germany).

### 2.5. Hematoxylin and Eosin (H&E) Staining

Colon tissue was initially fixed with 4% neutral buffered formalin and then graded dehydration with the ethanol solution (70%–100%) followed with xylene for 1 hr. Afterward, the tissues were embedded in the paraffin using a wax block. Subsequently, paraffin-embedded colon tissue was cut into thin sections of 5 *μ*M thickness using a rotary microtome (Leica Biosystems, Germany). Finally, the sliced sections were baked, deparaffinized, rehydrated, stained with H&E, and observed using the light microscope.

### 2.6. Real-Time Quantitative RNA (RT-qPCR)

Total RNA was extracted from colon tissues and macrophages of various groups using Trizol reagent (Invitrogen, CA, USA). Reverse transcription PCR was then carried out using real-time PCR (ABI 7500, CA, USA). The obtained values were normalized to the level of *GAPDH* mRNA and calculated using the 2^−*ΔΔ*Ct^ method.

### 2.7. Flow Cytometry

Surface markers of stimulated cells were detected using a flow cytometer. In short, peripheral blood mononuclear cells were isolated from each group of mice and incubated on ice with blocking antibody CD16/32 (Biolegend, San Diego, CA, USA) for 10 min. After washing the cells were incubated on ice with permeabilization reagent and anti-CD11b (Biolegend) in PBS for 30 min, avoiding light. After washing, the cells were suspended in 500 *μ*l PBS containing 3% FBS and then detected using flow cytometry (BD Biosciences, San Diego, CA, USA).

### 2.8. Statistical Analysis

In this study, all experimental data are analyzed by mean ± SD at least three independent experiments. The data analysis was performed using GraphPad Prism 9.0 software. The differences between the two groups were statistically analyzed using the student's *t*-test or one-way analysis of variance (ANOVA), considering *P* < 0.05 statistically significant.

## 3. Results

### 3.1. Effect of 5-MTP on DSS-Induced Colitis

**Figure 1:**
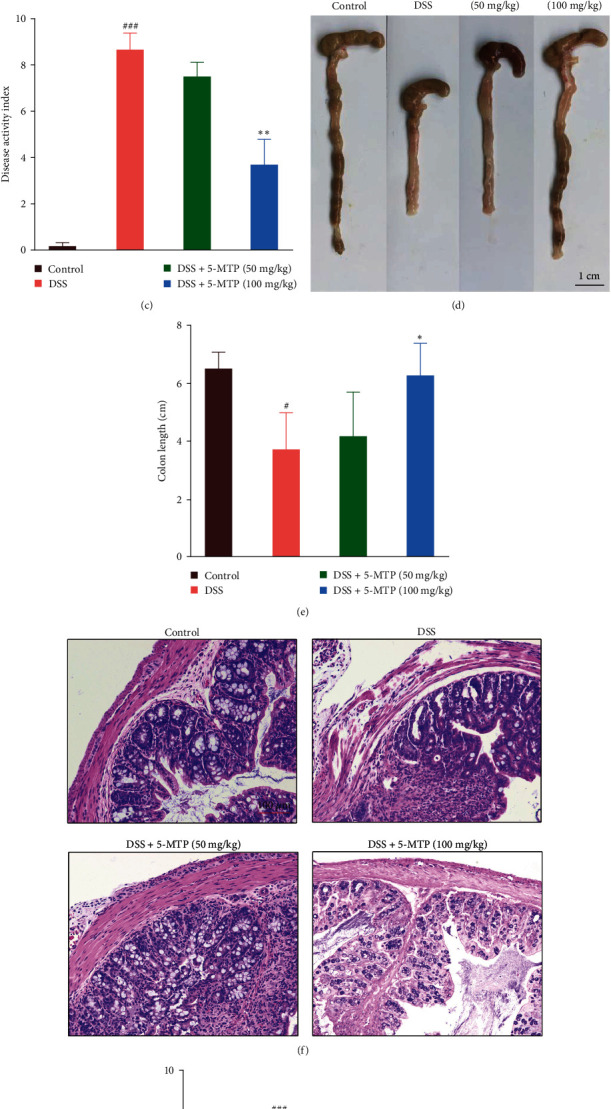
Effect of 5-MTP on DSS-induced inflammation in colon. (a) Experimental flowchart. (b) Body weights of mouse in each group (*n* = 5) were measured. The body weight was expressed as a percentage of weight change for each individual mouse and was calculated relative to the starting body weight on day 1 (*n* = 5). (c) Disease activity index (DAI) of mouse in each group (*n* = 5). (d) Macroscopic appearance of the representative colon from each group (*n* = 5). (e) The quantification of colon length from each group of mice (*n* = 5). (f) Histological changes of colon in each group were evaluated by H&E staining, scale bar: 100 *μ*m. (g) The quantification of colon histological score from each group of mice (*n* = 5). ^#^*P* < 0.05, ^##^*P* < 0.01, ^###^*P* < 0.001 compared with control group and  ^*∗*^*P*  < 0.05,  ^*∗∗*^*P*  < 0.01 compared with DSS-treated group.

As a methoxylation product of tryptophan metabolism, 5-MTP has emerged as one of the potential drugs derived from the tryptophan hydroxylase pathway. To demonstrate the effect of 5-MTP on the occurrence and treatment of colitis, we established a colitis mouse model induced by DSS (Figure 1(a)). As shown in Figure 1(b), compared with the control group, the average body weight of the mouse was significantly reduced after DSS induction for 7 days. Moreover, the weight loss of the mouse in the DSS induction treatment group persisted until day 14. In contrast, body weight loss in DSS-induced mouse was significantly reduced in the 5-MTP (both low and high dose) groups (Figure 1(b)). The DAI, a composite score, was recorded by measuring percent weight loss, stool consistency, and stool bleeding.  Figure 1(c) shows that the DAI score of the DSS-induced group was significantly increased, while the 5-MTP significantly alleviated the DAI of the mouse. Moreover, compared with the control group, the colon length of the DSS-induced group was significantly shortened, while the 5-MTP treatment group could significantly restore the colon length of the mouse. Specifically, it recovered to the colon length of the control group in the high-dose group (Figures 1(d) and 1(e)). Additionally, we also examined the morphology of colitis in different groups. Our research has shown that 5-MTP treatment group can alleviate the damage of DSS (Figures 1(f) and 1(g)).

Similarly, different concentrations (both low and high doses) of 5-MTP were able to alleviate TNBS-induced weight loss and DAI in mice (Figure S1). In addition, colon length was significantly restored in the TNBS-induced group in the 5-MTP treatment groups compared to the control group (Figures S1)). TNBS-induced intestinal damage was mitigated in the 5-MTP treatment groups (Figure S1). In summary, the DSS-induced mouse colitis model showed various apparent pathological indicators, which showed that 5-MTP displayed the therapeutic effect of relieving colitis *in vivo*.

### 3.2. Regulatory Effects of 5-MTP on Macrophage Infiltration and Inflammatory Cytokines in DSS-Induced Colitis *In Vivo*

**Figure 2:**
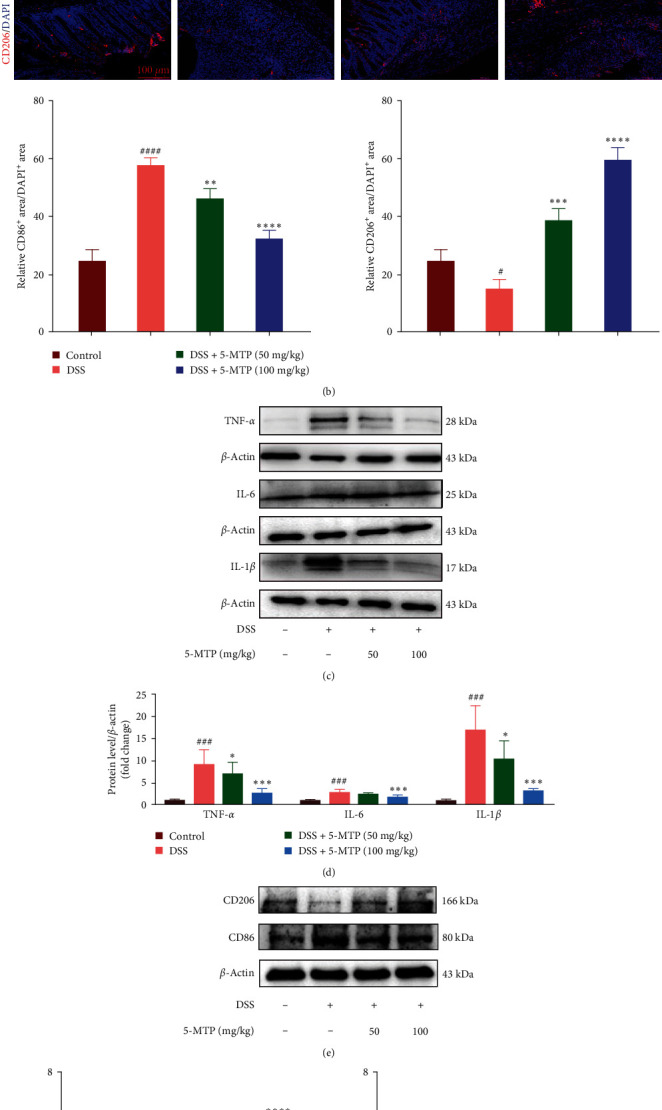
Regulatory effects of 5-MTP on macrophage infiltration and inflammatory cytokines in the colons of DSS-treated mouse. (a) The mRNA level of pro-inflammatory factor in each group was evaluated by RT-qPCR (*n* = 5). (b) Immunofluorescent pictures of CD86 and CD206-positvie cells in each group (*n* = 5), scale bar: 100 *μ*m (c) The protein expression levels of the pro-inflammatory cytokines were measured by western blotting. (d) The quantification of TNF-*α*, IL-6, and IL-1*β* expression in each group (*n* = 5). (e) The protein expression levels of the CD86 and CD206 were measured by western blotting. (f) The quantification of CD86 and CD206 expression in each group (*n* = 5). ^#^*P* < 0.05, ^##^*P* < 0.01, ^###^*P* < 0.001, and ^####^*P*  < 0.0001 compared with control group and  ^*∗*^*P*  < 0.05,  ^*∗∗*^*P*  < 0.01,  ^*∗∗∗*^*P*  < 0.001, and  ^*∗∗∗∗*^*P*  < 0.0001 compared with DSS-treated group.

In virtue of the aforementioned 5-MTP-based regulating effects on colitis and to assess the crucial role of 5-MTP in alleviating these responses, we further examined its effects on the infiltration of macrophages and the generation of various inflammatory factors. Here, we determined the phenotype of inflammatory cell infiltration in the colon tissues. As shown in Figure 2(a), 5-MTP treatment significantly contributed to the alleviation of the inflammatory factors, include TNF-*α*, IL-6, and IL-1*β*. The expression levels of the M1 macrophage marker CD86^+^ were notably elevated in the DSS-induced group. Conversely, treatment with 5-MTP significantly reduced CD86^+^ levels, with the high-dose treatment group's levels mirroring those of the control group. In contrast, CD206^+^, a marker for M2 macrophages, showed changes inversely proportional to those observed for CD86^+^ (Figure 2(b)). Additionally, we analyzed the protein expressions of proinflammatory cytokines in the colon tissues. In the DSS-induced group, the expressions of IL-1*β*, IL-6, and TNF-*α* were significantly higher than that in the control group. Contrarily, the levels of these proinflammatory cytokines in the 5-MTP-treated groups were significantly attenuated than that in the DSS-induced group (Figures 2(c) and 2(d)). In addition, 5-MTP significantly restored the decreased protein expression level of CD206 caused by DSS and increased the protein expression levels of CD86 induced by DSS (Figures 2(e) and 2(f)). These findings collectively indicate that 5-MTP effectively attenuates the expression of proinflammatory cytokines and facilitates the transition of macrophages from the M1 to the M2 phenotype.

### 3.3. Protective Effect of 5-MTP on Tight Junctions

**Figure 3:**
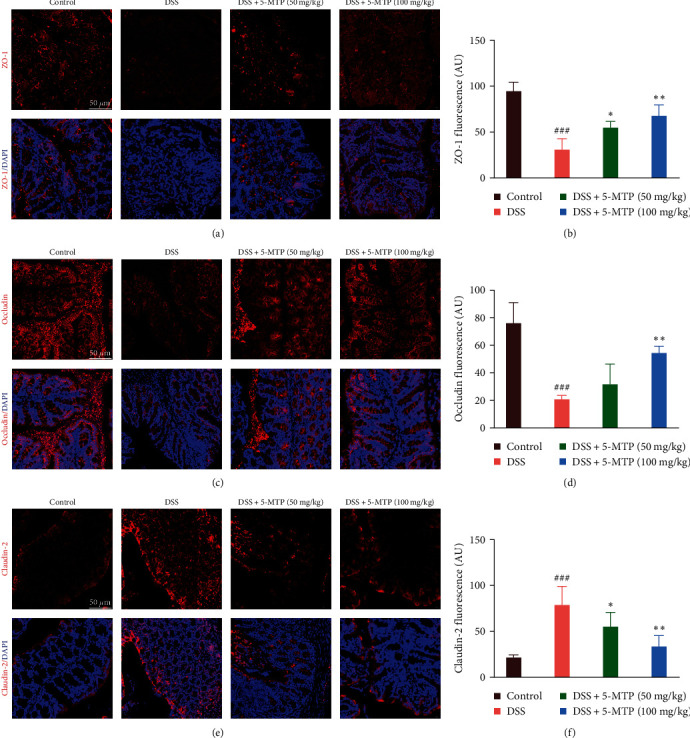
Protective effect of 5-MTP on tight junctions in the colons of DSS-treated mouse. (a, c, and e) Immunofluorescent pictures of ZO-1, occludin, and claudin-2 in the colon tissue, scale bar: 50 *μ*m. (b, d, and f) Quantitative graphs of ZO-1, occludin, and claudin-2 in the colon tissue in each group (*n* = 5). ^###^*P* < 0.001 compared with control group and  ^*∗*^*P* < 0.05,  ^*∗∗*^*P* < 0.01 compared with DSS-treated group.

The barrier functions of the gut include immune defenses, mucosal protections, and mechanical barriers. During an inflammatory reaction, the barrier function of the intestinal tract is initially damaged to different extents, which can progress to mechanical barrier damage, resulting in ulcers and potential leakage and perforation of the gut wall. Therefore, we initiated a study on the changes in proteins related to tight junctions that maintain the intestinal mechanical barrier. Here, our results show that the expressions of ZO-1 and occludin in the DSS-induced group were significantly decreased. Conversely, in the groups treated with varying concentrations of 5-MTP, the expression exhibited a dose-dependent increasing trend (Figures 3(a), 3(b), 3(c), and 3(d)). Surprisedly, the changing trend of claudin-2 was opposite to that of ZO-1 and occludin (Figures 3(e) and 3(f)). Above all, these results suggested that DSS substantially induced the damage to the intestinal tight junctions, while 5-MTP significantly alleviated this phenomenon.

### 3.4. Inhibitory Effect of 5-MTP on the Neutrophils in the Peripheral Blood and Tissue Apoptosis in DSS-Treated Mouse

**Figure 4:**
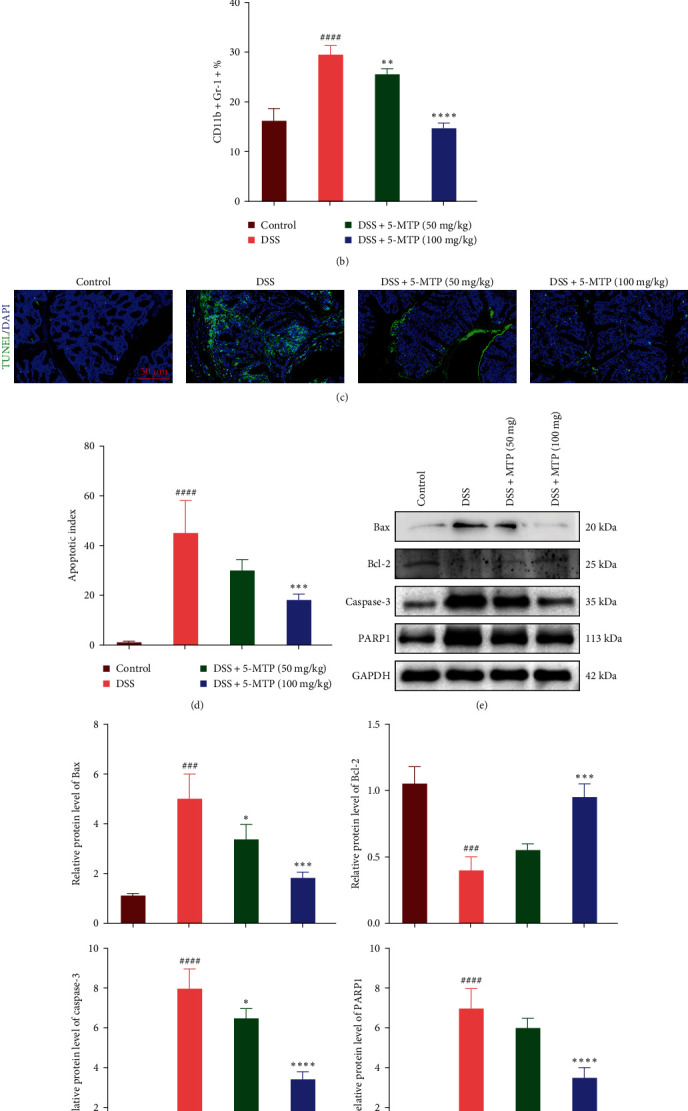
Inhibitory effect of 5-MTP on DSS-induced apoptosis in the colons of mouse. (a) The expression CD11b on the neutrophils in each group. (b) The quantification of CD11b in each group (*n* = 5). (c) TUNEL assay of colon tissue in each group. (d) Apoptotic index of each group (*n* = 5), scale bar: 50 *μ*m. (e) Protein expressions of Bax, Bcl2, caspase-3, and PARP1. (f) Quantification of each protein expression (*n* = 5). ^###^*P* < 0.001, ^####^*P* < 0.0001 compared with control group and  ^*∗*^*P*  < 0.05,  ^*∗∗*^*P*  < 0.01,  ^*∗∗∗*^*P*  < 0.001, and  ^*∗∗∗∗*^*P*  < 0.0001 compared with DSS-treated group.

As a marker of neutrophils, the level of CD11b^+^ neutrophils was upregulated in the DSS-induced group; however, the level of CD11b^+^ neutrophils was obviously reduced in the treatment of 5-MTP (Figures 4(a) and 4(b)). Indeed, the uncontrolled apoptosis of intestinal epithelial cells is one of the main phenomena of colitis. The generation of proinflammatory cytokines in response to colonic mucosal injury and immune abnormalities subsequently increases the apoptosis of intestinal epithelial cells, thereby aggravating the development of colitis. In this study, TUNEL staining results showed a large amount apoptotic cell in the colon of the DSS-induced group. In addition, the fluorescence intensity was significantly higher in the DSS-induced group than in the control group. On the contrary, the number of apoptotic cells was significantly reduced in the 5-MTP-treated groups with different concentrations, with the fluorescence intensities in the proximity of the control group (Figures 4(c) and 4(d). Simultaneously, we assessed the expression of crucial apoptotic markers, including Bax, Bcl-2, Caspase-3, and PARP1. The findings revealed a significant upregulation of Bcl-2 in the group treated with 5-MTP. Conversely, the expression levels of pro-apoptotic proteins such as Bax, Caspase-3, and PARP1 were markedly reduced. Collectively, these observations suggest that 5-MTP effectively diminishes apoptosis in intestinal epithelial cells (Figures 4(e) and 4(f).

### 3.5. 5-MTP Inhibits Inflammation and M1 Polarization Response of RAW264.7

**Figure 5:**
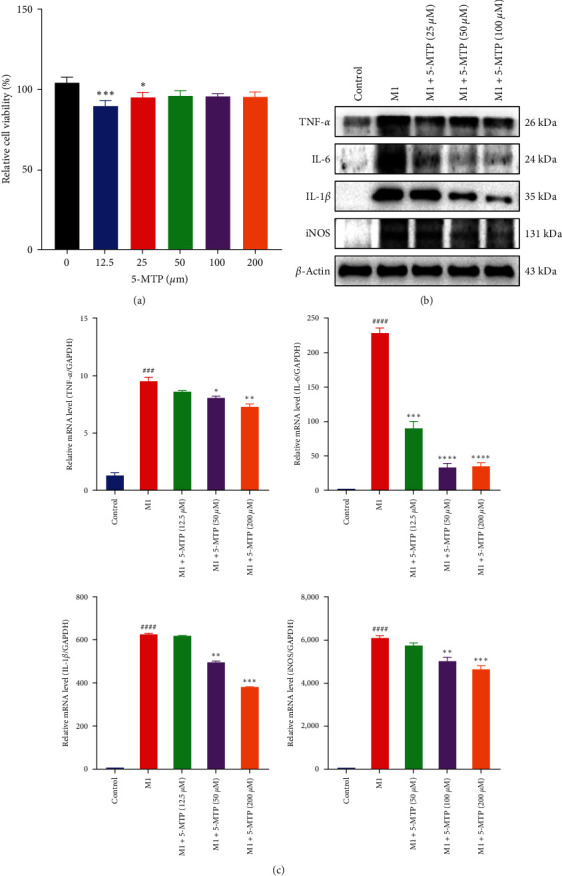
5-MTP inhibits inflammation and polarization response of RAW264.7. (a) The effect of 12.5, 25, 50, 100, and 200 *μ*M of 5-MTP on RAW264.7 cells was detected by CCK-8 assay after treatment for 24 hr. (b) Protein expression of TNF-*α*, IL-6, IL-1*β*, and iNOS in each group. (c) The mRNA level of TNF-*α*, IL-6, IL-1*β*, and iNOS in each group. ^###^*P* < 0.001 and ^####^*P* < 0.0001 compared with control group and  ^*∗*^*P*  < 0.05,  ^*∗∗*^*P*  < 0.01,  ^*∗∗∗*^*P*  < 0.001, and  ^*∗∗∗∗*^*P*  < 0.0001 compared with DSS-treated group.

In our research, CCK-8 assays revealed that at concentrations below 100 *μ*M, 5-MTP significantly promoted cell proliferation in dose-dependent fashion after cultured for 24 hr (Figure 5(a)). Therefore, the concentrations of 25, 50, and 100 *μ*M of 5-MTP were used for further study. Moreover, the expression of IL-1*β*, IL-6, TNF-*α*, and iNOS in M1 macrophage cell were significantly increased. In contrast, their expressions in the treatment groups with different concentrations of 5-MTP were reduced in a dose-dependent manner (Figure 5(b)). Additionally, the mRNA levels of IL-1*β*, IL-6, TNF-*α*, and iNOS were upregulated in M1 macrophage, whereas they were considerably decreased in the 5-MTP treatment groups (Figure 5(c)). These results demonstrated that 5-MTP effectively reduced the expression of related pro-inflammatory cytokines and M1 polarization response of RAW264.7 *in vitro*.

## 4. Discussion

5-MTP, a known tryptophan metabolite, regulates numerous cellular processes, including the regulation of inflammatory response [15]. In addition to participating in various physiological and pathological processes of cancer, 5-MTP plays a critical role in the remission of various inflammatory diseases [11]. Considering these aspects, this study revealed the potential role of 5-MTP in the alleviating and treating colitis, a refractory intestinal inflammatory disease. Our findings demonstrated that 5-MTP could inhibit macrophage infiltration and generation of various inflammatory factors in a DSS-induced colitis mouse model. In addition, 5-MTP inhibited tight junction damage and apoptosis of the intestinal epithelial cells. 5-MTP effectively reduced the expression of pertinent pro-inflammatory cytokines and M1 polarization response *in vitro*, which suggested that 5-MTP displayed an excellent therapeutic effect against colitis, broadening its application scope of anti-inflammatory effect.

Indeed, this tryptophan metabolite is known to offer a relieving effect on the inflammatory response in the body [16]. In this study, we found that 5-MTP could significantly alleviate the infiltration of macrophages and the release of inflammatory factors in a DSS-induced colitis mouse model. Generally, macrophages play a pivotal role in mediating inflammation. Their activation triggers the synthesis and release of a wide array of proinflammatory cytokines and chemokines, significantly driving severe inflammatory responses and leading to extensive tissue damage [17, 18, 19]. Previous reports indicated that 5-MTP alleviated septic organ failure by targeting macrophages and transcriptionally inhibited LPS-induced expressions of IL-1*β*, TNF-*α*, and IL-6 in mouse peritoneal macrophages [11]. Notably, the therapeutic inhibitory effect of 5-MTP on macrophages was consistent with our findings. In addition to the effects mentioned above on macrophage infiltration, our results show that 5-MTP could reduce other mediators, such as TNF-*α*, IL-6, and IL-1*β*. The release of 5-MTP indicated that the anti-inflammatory effect in the colitis animal model was consistent with that in the septic systemic inflammation animal model. Despite the success in exploring its effect on inflammatory mediators in the colitis model, the mechanistic effect of 5-MTP either through the NF-*κ*B signaling pathway or the transcription of COX-2 needs to be further studied. Notably, the therapeutic inhibitory effect of 5-MTP on macrophages was consistent with our findings. In addition to the effects mentioned above on macrophage infiltration, our results shown that 5-MTP could reduce other mediators, such as TNF-*α*, IL-6, and IL-1*β*. The release of 5-MTP indicated that the anti-inflammatory effect in the colitis animal model was consistent with that in the septic systemic inflammation animal model. Despite the success in exploring its effect on inflammatory mediators in the colitis model, the mechanistic effect of 5-MTP either through the NF-*κ*B signaling pathway or the transcription of COX-2 needs to be further studied.

In addition to the effect of 5-MTP on macrophages in the inflammatory response, this investigation showed that 5-MTP displayed a notable alleviating effect on the inflammatory damage to the intestinal epithelium [20]. Several *in vitro* reports demonstrated that 5-MTP could inhibit the degradation of VE-cadherin in human umbilical vein endothelial cells (HUVECs), thereby protecting Endothelial cells (ECs) from lipopolysaccharide (LPS) and cytokine-induced barrier damage and ultimately maintaining the tight endothelial junctions [13]. Similar to the tight junction barrier of vascular ECs, intestinal epithelial cells are highly polarized cells. The barrier formed by tight junctions is a key structural basis for maintaining normal intestinal function. In this study, we have shown that 5-MTP could significantly alleviate the changes in the expression of tight junction proteins, such as ZO-1, occludin, and claudin-2 in DSS-induced intestinal epithelial tissues, thereby reducing the damage to the intestinal epithelium caused by the generated inflammatory responses. Some contrary findings in other studies indicated that 5-MTP could act on tight endothelial junctions through the p38 mitogen-activated protein kinase (MAPK) pathway. This key signaling pathway disrupted endothelial barrier function and guided leukocyte adhesion and migration [21, 22]. Remarkably, our research highlights that the influence of 5-MTP on the expression of key tight junction proteins, including ZO-1, occludin, and claudin-2, potentially involves certain signaling pathways. This observation underscores the need for further detailed investigations to elucidate the underlying mechanisms.

In view of the alleviating effect on intestinal epithelial injury, we further explored the inhibitory effect of 5-MTP on the apoptosis of epithelial cells. Notably, the protective effect of 5-MTP on the intestinal epithelium damaged by inflammation is further elaborated. Studies have reported that intestinal epithelial cells can experience inflammation and apoptosis due to DSS, which can consequently contribute to the development and advancement of colitis [23, 24]. Mitigating DSS-induced inflammation can be accomplished through the inhibition of apoptosis. In investigating the impact of 5-MTP on the apoptosis of intestinal epithelial cells, we observed alterations in the expression of key pro-apoptotic and antiapoptotic proteins, including caspase-3, PARP, Bax, and Bcl-2. Furthermore, the role of autophagy-induced apoptosis in this context remains to be elucidated, marking a critical avenue for our future research endeavors.

## 5. Conclusion

Our research indicated that 5-MTP inhibits the infiltration of macrophages and the generation of inflammatory factors, both *in vivo* and *in vitro*. In addition to its effects on immune cells, 5-MTP can significantly suppress tight junction damage and apoptosis in intestinal epithelial cells *in vivo*. Our research suggests that 5-MTP may be a potential therapeutic drug for colitis, which could offer new ideas and strategies for the treatment of inflammatory bowel disease, with the potential to improve the efficacy and safety of current treatment regimens. This study deepens our understanding of the pathogenesis of inflammatory bowel disease and provides a wider range of therapeutic options for the treatment of inflammatory bowel disease and even other inflammation-related diseases.

## Data Availability

All data are provided in this study, and raw data can be requested to the corresponding author.

## Abbreviations

5-MTP: 5-methoxytryptophan
TNBS: 2,4,6-trinitrobenzenesufonic acid
H&E: Hematoxylin and eosin
TUNEL: Terminal deoxynucleotidyl transferase dUTP nick end labeling
ECs: Endothelial cells
DAI: Disease activity index
IACUC: Institutional Animal Care and Use Committee
RT-qPCR: Real-time quantitative RNA
HUVECs: Human umbilical vein endothelial cells
ECs: Endothelial cells
LPS: Lipopolysaccharide
MAPK: Mitogen-activated protein kinase.

## Supplementary Materials

Supplementary Materials Figure S1: effect of 5-MTP on TNBS-induced inflammation in colon.
