# Supplementary material for: 5-Methoxytryptophan Alleviates Dextran Sulfate Sodium-Induced Colitis by Inhibiting the Intestinal Epithelial Damage and Inflammatory Response
Source: Mediators Inflamm. 2024 Jul 17;2024:1484806. doi: 10.1155/2024/1484806 (PMC11390199; doi:10.1155/2024/1484806)
Supplement: Supplementary Materials — Figure S1: effect of 5-MTP on TNBS-induced inflammation in colon. [file 1484806.f1.docx]

**5-methoxytryptophan alleviates dextran sulfate sodium-induced colitis by inhibiting the intestinal epithelial damage and inflammation responses**

Yanling Wang^1#^, Jun Li^1#^, Qinyuan Yang^2#^, Zhenhang Zhu^1^, Fang Cheng^1^, Xiangyan Ai^1^, Yang Liu^1^, Dongbao Zhao^5*^, Peng Cheng^3, 4*^, Futao Zhao^1*^

*^1^Department of Rheumatology and Immunology, Shanghai Ninth People's Hospital, Shanghai Jiao Tong University School of Medicine, Shanghai, China;*

*^2^Department of Geriatrics, Huadong Sanatorium, Wuxi, Jiangsu 214000, China;*

*^3^Department of Gastroenterology, Hainan West Central Hospital, 2 Fubo East Road, Danzhou, Hainan, China;*

*^4^Department of Gastroenterology, Shanghai Ninth People's Hospital, Shanghai Jiao Tong University School of Medicine, No. 639 Zhizaoju Road, Shanghai, China;*

*^5^Department of Rheumatology and Immunology, Changhai Hospital, Naval Medical University, Shanghai 200433, China;*

#These authors contributed equally to this work.

**Corresponding authors:**

Futao Zhao, E-mail: [72300611413@shsmu.edu.cn;](mailto:72300611413@shsmu.edu.cn;) Peng Cheng, E-mail: [imettoo@163.com;](mailto:imettoo@163.com;) Dongbao Zhao, E-mail: dongbaozhao@163.com;

**
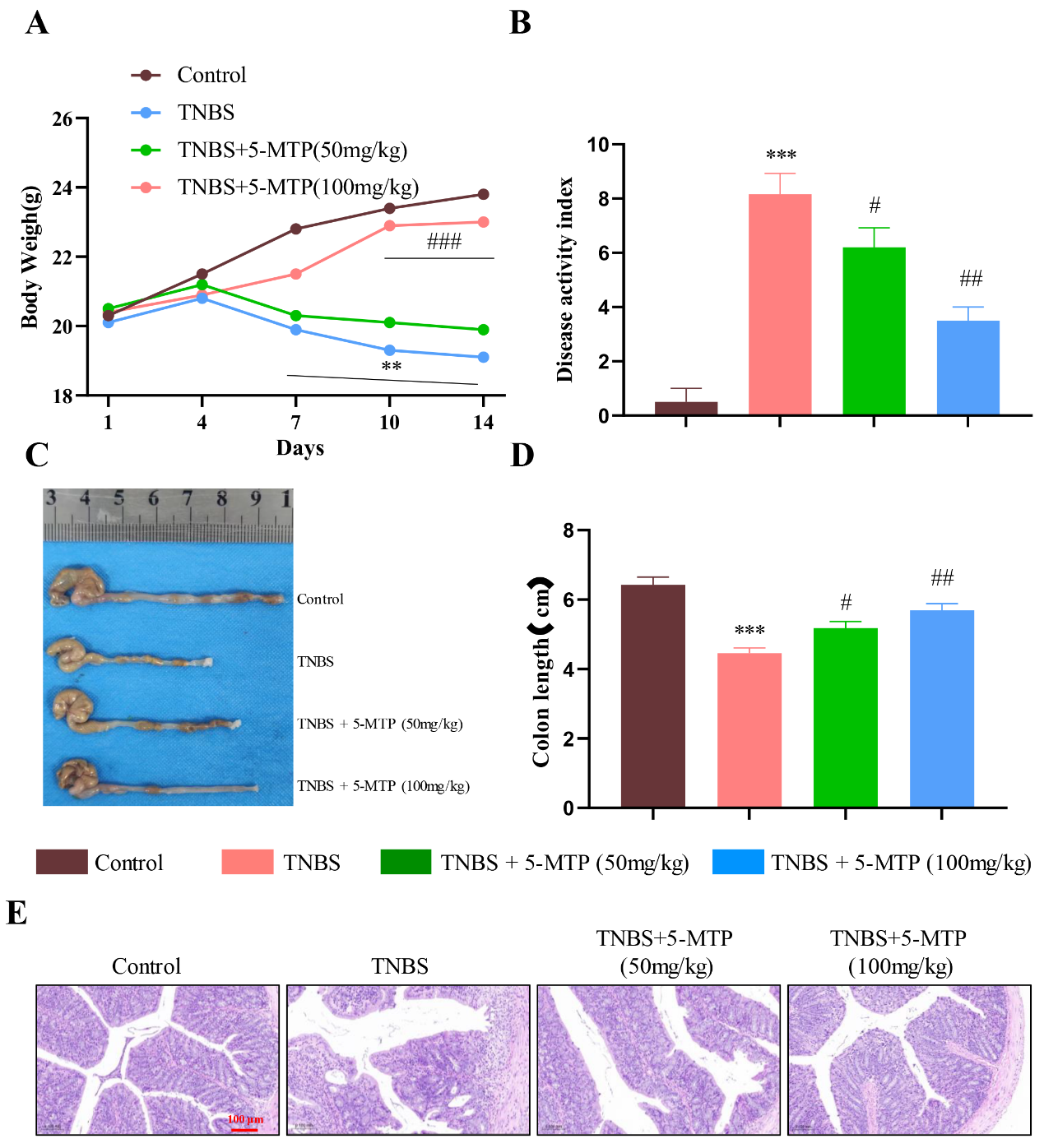
**

**Supplementary Figure 1. Effect of 5-MTP on TNBS-induced inflammation in colon. A.** Body weights of mouse in each group (n = 5) were measured. **B.** Disease activity index (DAI) of mouse in each group (n = 5). **C.** Macroscopic appearance of the representative colon from each group (n = 5). **D.** The quantification of colon length from each group of mice (n = 5), scale bar: 100 μm. **E.** The quantification of colon histological score from each group of mice (n = 5). *^**^P* < 0.01, *^***^P* < 0.001 compared with control group and *^#^P* < 0.05, *^##^P* < 0.01, *^***^P* < 0.001 compared with TNBS-treated group.
